# A Review of Gene Delivery and Stem Cell Based Therapies for Regenerating Inner Ear Hair Cells

**DOI:** 10.3390/jfb2030249

**Published:** 2011-09-13

**Authors:** Keerthana Devarajan, Hinrich Staecker, Michael S. Detamore

**Affiliations:** 1 Bioengineering Program, University of Kansas, Lawrence, KS 66045, USA; E-Mail: keerthanad@gmail.com; 2 Department of Otolaryngology Head and Neck Surgery, University of Kansas School of Medicine, Kansas City, KS 66160, USA; E-Mail: hstaecker@kumc.edu; 3 Department of Chemical and Petroleum Engineering, University of Kansas, Lawrence, KS 66045, USA

**Keywords:** sensory neural hearing loss, vestibular dysfunction, hair cell regeneration, viral vectors, gene delivery, stem cells

## Abstract

Sensory neural hearing loss and vestibular dysfunction have become the most common forms of sensory defects, affecting millions of people worldwide. Developing effective therapies to restore hearing loss is challenging, owing to the limited regenerative capacity of the inner ear hair cells. With recent advances in understanding the developmental biology of mammalian and non-mammalian hair cells a variety of strategies have emerged to restore lost hair cells are being developed. Two predominant strategies have developed to restore hair cells: transfer of genes responsible for hair cell genesis and replacement of missing cells via transfer of stem cells. In this review article, we evaluate the use of several genes involved in hair cell regeneration, the advantages and disadvantages of the different viral vectors employed in inner ear gene delivery and the insights gained from the use of embryonic, adult and induced pluripotent stem cells in generating inner ear hair cells. Understanding the role of genes, vectors and stem cells in therapeutic strategies led us to explore potential solutions to overcome the limitations associated with their use in hair cell regeneration.

## 1. Introduction

Hearing loss has become one of the most common disabilities in the United States and can affect almost every age group. The number of people with hearing loss worldwide has been steadily increasing over recent years, reaching almost 49 million people in the US alone [1]. According to the National Institute on Deafness and Other Communication Disorders (NIDCD) 2010 statistics, approximately 17% of the American adult population experiences hearing loss and 3 out of every 1000 children are born deaf. The prevalence of hearing loss increases with age, as about 47% of adults over 75 years old have hearing impairment (NIDCD). Although hearing loss may not be life threatening, it can greatly influence the patient's quality of life, social interactions, and have a significant financial impact on society [1,2].

The ear is of tremendous importance in sensing the world around us. Aside from being the prime organ for the perception of sound, it also plays a crucial role in balance. The inner ear is a highly specialized sense organ with a complex structure and has been referred to as a labyrinth [3]. It contains hair cells arranged in a highly organized pattern and is innervated by sensory neurons. Acoustic energy, in the form of sound waves, is channeled into the ear canal where it strikes the tympanic membrane. As the energy hits the stapes located at the oval window, a pressure wave sets the cochlear fluid into motion. The hair bundles deflect as a result of the shearing force caused by the cochlear fluid and the stereocilia slide along one another. The movement on the stereocilia results in their depolarization and opens up gated calcium ion channels. This is followed by the release of neurotransmitters from the base of the hair cell. Primary auditory afferents then conduct the signal to the brainstem.

Sensorineural hearing loss (SNHL) involves damage to the cochlea (inner ear sensory hair cells) or the eighth nerve. It is irreversible and in most cases a hearing aid is required. Common causes for SNHL are aging, ototoxic drugs, noise induced trauma, inner ear concussion, and immune disorders [4,5,6]. Although only a small percentage of the cases can be treated medically and surgically, advances in molecular and stem cell therapies may provide tools to treat the irreparable damage of hair cells caused by SNHL [7].

Hair cells are sensory receptors located in the inner ear. They appear to be “hair-like” because of the numerous ciliary processes called stereocilia that extend from their surfaces. Hair cells are responsible for converting sound into electrical signals that are sent to the brain via the auditory nerve for processing. In the human cochlea, hair cell death can occur due to a variety of causes, such as age related deafness (presbycusis), a high dosage of ototoxic drugs (e.g., gentamycin, cisplatin, aminoglycosides), genetic disorders, infectious diseases, or high levels of noise exposure [3,8,9]. Patients exposed to high doses of aminoglycosides as a treatment regimen for bacterial infections often experience hair cell death. The aminoglycosides are known to induce cell death via activation of the intracellular caspase signaling pathway and trigger mitochondria to release cytochrome c into the cytoplasm, which consequently induces apoptosis via caspase-3 activation in hair cells. Noise induced cellular stress activates the JNK signaling pathway and causes neuronal cell death via necrosis. Necrotic hair cell death is less common than apoptotic cell death and is mainly induced by trauma or disease [3,9,10].

Over the past 30 years, several attempts have been made to understand the pathways involved in hair cell formation and death [11]. The process of hair cell regeneration was considered impossible to occur in higher vertebrates until two groups serendipitously discovered the amazing phenomenon in birds in the late 1980s [12]. At the same time, Jørgensen and Mathiesen showed that there were mitotic activity and continuous proliferation of hair cells in the vestibular epithelium of adult parakeets [13]. Ever since, there has been steady progress in understanding the mechanics and pathways of avian and mammalian (rat, mouse, guinea pig) hair cell regeneration [8,10,14,15,16,17,18,19,20,21]. Significant development in hair cell regeneration is outlined in the following reviews [7,22,23,24,25,26,27,28,29]. In this review, we explore how different genes, viral vectors and stem cell sources can be used in conjunction with each other to engineer inner ear hair cells and develop strategies in restoring the function of the inner ear.

## 2. Hair Cells, Supporting Cells and Their Function

### 2.1. Hair Cells

**Figure 1:**
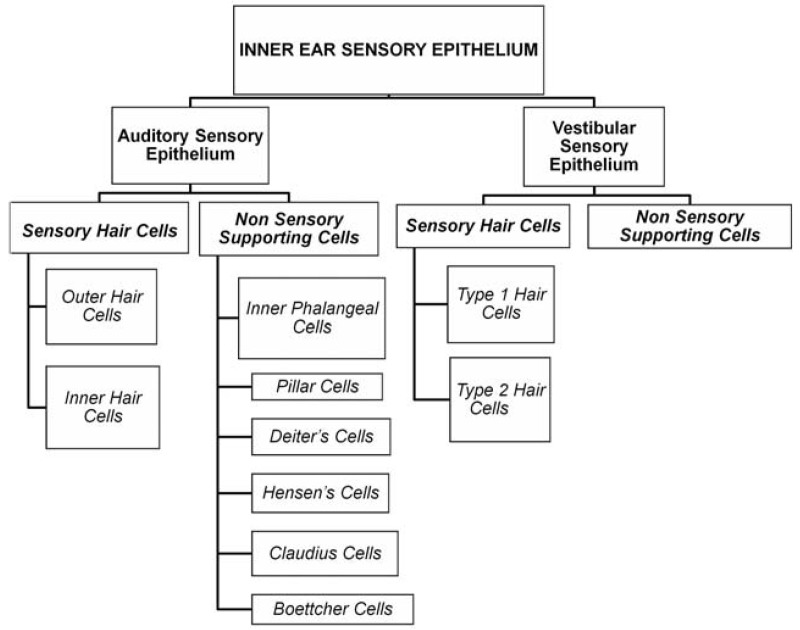
Classification of inner ear sensory epithelium.

Hair cells are the mechanoreceptors in the inner ear that detect sound, head movements and orientation in space [9,30]. They are formed in the embryonic stages during the development of the inner ear. The otic ectoderm undergoes proliferation and gives rise to the sensory primordium. The proliferating sensory primordium precursors form the organ of corti from within the cochlear epithelium and patterns into an organized array of inner and outer hair cells separated from each other by non-sensory supporting cells. Hair cells are present in the sensory epithelium of both the auditory (inner and outer hair cells) and vestibular systems (Type 1 and Type 2 hair cells). All hair cells are surrounded by supporting cells and have physiological and morphological differences. Figure 1 shows the classification of inner ear sensory epithelium. Sensory epithelium in the inner ear is composed of both sensory and non-sensory cell types that have morphological and physiological differences. Table 1 gives the differences between inner and outer hair cells.

**Table 1:** Differences between inner and outer hair cells.

**Factor**	**Inner hair cells**	**Outer hair cells**	**Ref.**
**Arrangement**	Arranged in a single row	Arranged in three parallel rows	[31,32,33]
**Shape**	Round and small	Long and slim	[10]
**Function**	Transduce mechanical energy to neural signals	Appear to impact and regulate the sensitivity of the cochlea over a range of 32 dB	[10]
**Effects**	Sensory Neural Hearing loss	Alter properties of cochlear input to the brain	[34]
**Approximate number**	3,000 to 3,500	9,000 to 12,000	[32,33]

#### 2.1.1. Hair Cell Structure

**Figure 2:**
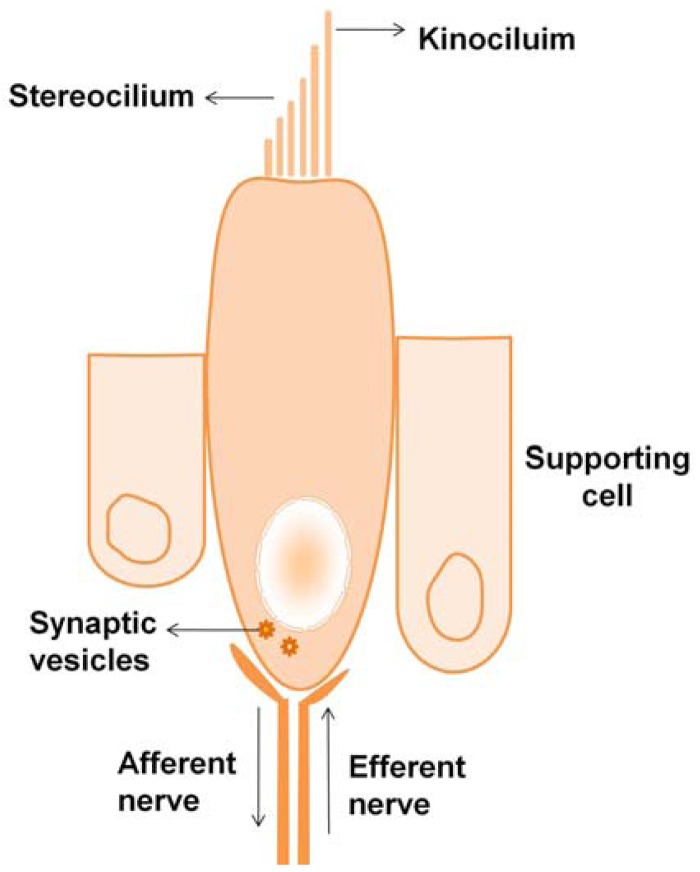
Structure of a typical vestibular hair cell.

Hair cells are flask-shaped cells with extended processes at their apical ends called stereocilia. The stereocilia are made of actin filaments and actin bundling proteins—fimbrin and espin [35]. The vestibular system hair cells have one true cilium called the kinocilium that is thought to mark the polarity of the cell. Figure 2 shows the schematic representation of a type 2 vestibular hair cell, showing the flask-shaped hair cell supported by supporting cells at the base. The stereocilia at the top are connected by filamentous tip links that participate in transduction and ion exchange.

The kinocilium of cochlear hair cells in mammals degenerates a few weeks after birth [36,37,38,39,40]. Hair cells form synaptic connections from neurons at the basal end and terminate in the cochlear or vestibular nuclei of the brainstem. The term “mechanoreceptors” comes from their participation in transforming mechanical energy to electrical energy. In the auditory system, the mechanical energy occurs in the form of a wave and the hair cells are stimulated as the tectorial membrane shears against the organ of corti, whereas in the vestibular system, the mechanical energy is a result of displacement of hair cells due to the force of gravity or inertia.

#### 2.1.2. Transduction in Hair Cells

The stereocilia are connected together by a filamentous tip link. The tips are connected to cation transduction channels that are involved in calcium and potassium ion exchange. Each hair cell has about 100 transduction channels; the hair cell's mechanical movement and ion exchange control the cell's membrane potential and provide the driving force for auditory or vestibular nerve excitation. Myosin motor proteins are activated by the calcium ions and play an important role in triggering the hair cell's adaptation to mechanical stimuli from stereocilia deflections [11].

### 2.2. Supporting Cells

Supporting cells are the non-sensory cells of the sensory epithelium and do not take part in sound transduction. They are located at the base of the hair cells and surround them, preventing contact between individual hair cells. The mammalian auditory system has two main types of supporting cells: the Deiter's cells support the outer hair cells at the base, and the pillar cells help in forming the reticular lamina, which isolates the stereocilia from their cell bodies. A few other supporting cells types include innerphalangeal cells, Hensen's cells, Claudius cells and Boettcher cells that are specialized cells and are not associated with hair cells [41]. Supporting cells of the vestibular system have yet to be studied in detail to understand their physiological and morphological differences [24,30]. Supporting cells are known to play an important role in avian hair cell regeneration by two different mechanisms: mitotic regeneration and transdifferentiation [42]. The former occurs when the supporting cell divides mitotically, stimulating one of them to differentiate into a hair cell, whereas the latter occurs when a supporting cell changes its gene expression and becomes a hair cell directly without dividing. Healthy supporting cell populations are vital for a number of hair cell regeneration strategies that rely on transdifferentiation to restore the damaged neuroeptithelium.

### 2.3. Summary

Hair cells reside in the auditory and vestibular systems and are responsible for mechanoelectrical transduction of sound. Ototoxic drugs or noise induced stress can damage hair cells, thus compromising inner ear function. Supporting cells located at the base of the hair cells have the capability to regenerate hair cells in the vestibular system by mitotic division or transdifferentiation, though supporting cell proliferation may not always occur to replace lost hair cells.

## 3. Essential Genes in Hair Cell Differentiation

It is important to identify and characterize genes that govern the ontogeny and differentiation of the cochlear epithelium; identifying such genes can lead to the design of several therapeutic approaches for sensory epithelial cell development and hair cell differentiation. A critical gene responsible for inner ear development is the *Atonal* gene—a protein belonging to the basic helix-loop-helix (bHLH) family of transcription factors that activates the E-box dependent transcription. Atoh1 has a unique auto regulatory enhancer element containing an E-box in the 3′ region of the gene [43]. *Math1*, also known as *Atoh1*, is the mouse homolog of the *Drosophila melanogaster Atonal* gene. The *Math1* gene is essential for the differentiation of sensory hair cells from previously established sensory primordium and is limited to only a subpopulation of the non-sensory supporting cells, primarily the pillar cells [44,45]. Studies with embryonic *Math1*-null mice reported a failure to produce hair cells and deletion of *Atoh1* using Pax2-*Cre* resulted in degeneration of cells in the organ of corti in mice [46], proving *Math1* as a positive regulator in directing hair cell differentiation [47]. Gene delivery studies in guinea pigs, mice, and rats reported an over expression of *Math1* in non-sensory cells, resulting in the production of ectopic immature hair cells outside the sensory epithelium via the transdifferentiation mechanism [16,44,48,49,50,51,52]. The non-sensory *Math1* expressing cells attracted auditory nerve fibers and developed into mature hair cells [49,50].

The other homologues of the *Atonal* gene are *Cath1* (chicken atonal homolog), *Xath1* (Xenopus atonal homolog) and *Hath1* (human atonal homolog), although *Math1* is the most extensively studied and used transcription factor [53,54]. Studies with adenoviral expression of *Hath1* in rats showed hair cell production without supporting cell proliferation [55].

Additional genes involved in the control of supporting cell fate include *Hes1*, *Hes5*, *BETA2/Neurod1*, *Jagged2* and Notch Signaling [18,19]. *Hes1 and Hes5* have been shown to influence supporting cell fate through negative regulation of *Math1* [56,57]. Certain cell cycle kinases also influence inner ear development by regulating cell cycle and inhibiting hair cell differentiation (Refer Table 2). *BETA2/Neurod1* gene has been shown to regulate the formation of sensory and neuronal ganglions in both cochlear and vestibular systems [58]. Table 2 gives a list of the different genes involved in hair cell differentiation.

**Table 2:** Summary of different genes used in inner ear gene therapy.

**Gene**	**Role**	**Reference**
*Math1*	Also known as *Atoh1*. Primary gene responsible for hair cell differentiation. Other homologues include *Hath1*, *Cath1* and *Xath1*.	[47,49,53,54,55]
*Hes1* and *Hes5*	Mammalian homologues of *Hairy* and *Enhancer-of-split* gene. Expressed in supporting cells and known to be negative regulators of *Math1*. However, a balance between *Hes1/Hes5* is required to control the production of supernumerary hair cells and normal development of inner ear.	[56,57]
*Sox2*	Responsible for development of inner ear sensory epithelium and is expressed in supporting cells and inner ear progenitors. Acts upstream of *Math1* and maintains mitotic and transdifferentiation functions of supporting cells.	[59,60,61]
*Jag2*	Member of the notch signaling pathway. Expressed in supporting cells of auditory and vestibular system. Required for the normal development of inner ear sensory organs.	[19,61,62]
*BETA2/Neurod1*	Expressed in neurons and neural precursor cells. Promotes the formation of ganglion neurons in the cochlea. Absence of *BETA2/NeuroD* can compromise hair cell function. It is known to differentiate outer hair cells to inner ear hair cells and neurons to hair cells.	[58,63]
Rb1/Rbl2	Required for hair cell quiescence and cell-cycle exit of embryonic mammalian hair cells but not for their early differentiation. Deletion of Rb1 from progenitor cells leads to aberrant hair cell and supporting cell. Deletion of Rbl2 results in extra row of hair cells and supporting cells in apical regions of the cochlea.	[64,65,66,67,68]
Cdkn1b and Cdkn2d	Cyclin-dependent kinase inhibitor. Expressed in sensory progenitors during the early embryonic development of the cochlea. Regulates cell cycle and inhibits hair cell differentiation.	[69,70,71]
MYCN	Member of the *Myc* family that regulates proliferation. Regulates the growth of the ear as a whole and promotes differentiation of certain sensory and non-sensory components. Absence of MYCN can lead to abnormal development of inner ear organs and disorganized neuronal innervations.	[72]

**Figure 3:**
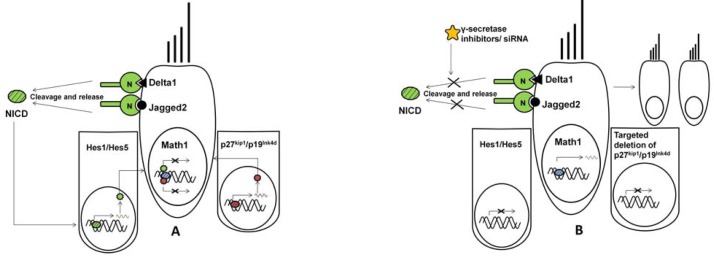
Schematic on the interaction of different genes and their contribution to positive and negative regulation of *Math1* transcription factor.

Figure 3 represents a schematic on the interaction of different genes and their contribution to positive and negative regulation of *Math1* transcription factor in neonates and during the embryonic development of the cochlea. (A) Hair cells express hair cell-specific *Math1* transcription factor and notch ligands—*Delta1* and *Jagged2*. Notch receptor (N) binds to the ligands, cleaves with the help of γ-secretase and releases Notch Intracellular Domain (NICD). NICD enters the nucleus of supporting cells and activated *Hes1/Hes5* transcription factors. *Hes1/Hes5* proteins inhibit *Math1* gene expression. Alternatively, expression of Cdkn1b (p27^kip1^) and Cdkn2d (p19^Ink4d^) in early progenitor supporting cells repress *Math1* expression and maintain supporting cell fate. (B) In the presence of γ-secretase inhibitors, the notch receptor fails to cleave and release the NICD, thus inhibiting the activation of *Hes1/Hes5* that would otherwise down regulate *Math1* expression. Similarly, targeted deletion of p27^kip1^ and p19^Ink4d^ genes allows ectopic expression of *Math1* resulting in supernumerary hair cells. These pathways can be induced or inhibited via standard or molecular therapy and additionally can be used to control the differentiation of stem cells.

## 4. Gene Therapy and Stem Cell-Based Approaches for Treatment of Sensory Neural Hearing Loss

Current therapies for treating hearing loss involve the use of either hearing aids or cochlear implants. Cochlear implants are only available to patients with severe hair cell damage and profound loss of hearing ability. However, the implants are not absolutely efficient in restoring hearing; their performance varies from patient to patient and requires training to adapt to the device. With advances in regenerative medicine using stem cells and gene therapy, several new strategies have emerged with the hope of permanently curing deafness. Some of these strategies are discussed in the following paragraphs.

### 4.1. Gene Therapy in the Inner Ear

A key to modifying cell phenotype is developing an effective means of delivering genes to the inner ear. As discussed earlier, the *Math1* gene is an essential gene in the differentiation of hair cells. Multiple approaches to deliver the *Math1* gene into the inner ear have been evaluated. Most of the approaches involve the injection of viral or non-viral vectors into the inner ear canal to trigger endogenous cells in the organ of corti to differentiate. There are a number of other popular routes of vector administration for inner ear gene therapy that are well explained in the literature [73,74,75,76].

An ideal vector is one that would ensure patient safety and effective transformation of undifferentiated cells. The commonly used vectors are derived from adenovirus, adeno-associated virus (AAV), herpes virus and lentivirus. Adenoviral mediated *Math1* and *Hath1* gene delivery has shown promising results *in vivo* for regenerating inner ear hair cells in mammals [16,48,49,51,77]. Adenoviral vectors offer high transfection efficiency and have been extensively investigated in clinical trials for ocular disease and cystic fibrosis [78,79]. The prior experience with adenovectors in the clinic can make them more desirable for commercial and clinical applications. The efficacy of adenoviral vectors can be improved by modifying vector elements, such as using tissue specific promoters or deleting DNA sequences to eliminate production of harmful viral proteins. Adenoviral mediated vestibular hair cell regeneration has been reported to be more efficient with the glial fibrillary acidic protein (GFAP) promoter than the human cytomegalovirus (hCMV) and chicken β actin (cBA) promoters. Additionally, adenovectors with deleted E4 regions resulted in increased tolerability of the cells toward the vector [51,52,80]. In addition to delivering the *Math1* gene, adenoviral vectors are used to deliver growth factors such as transforming growth factor-β1 (TGF-β1), glial cell-derived neurotrophic factor (GDNF), and B-cell lymphoma 2 (Bcl-2) to protect hair cells from sound trauma and ototoxic drugs [81,82]. Despite the success of adenoviruses in the effective delivery of *Math1*, short term gene expression and strong immune responses often result, but can be overcome by adeno-associated viruses. Studies show the capacity of adeno-associated viruses to deliver genes to inner ear blood vessels and certain auditory nerve fibers with negligible toxicity when compared to adenoviruses [76,83]. *Herpes simplex* viral vectors (HSV) derived from *Herpes simplex type I* can infect and replicate in non-dividing cells. Studies in cochlear gene therapy using *HSV* reported dispersed gene expression in the cochlea, limited to auditory and vestibular spiral ganglion neurons [84]. A limitation of *HSV* mediated gene delivery is that it requires the use of high viral stock volumes due to the difficulty of producing the vectors in high titers. In addition, *HSV* are reported to evoke strong inflammatory responses in guinea pigs [85]. Lentivirus is the best available viral vector in terms of transduction efficiency and transgene expression because of its ability to infect both proliferating and non-proliferating cells, including stem cells that are difficult to transduce. Research in lentiviral-mediated gene delivery in the guinea pig cochlea showed gene expression limited to the perilymphatic space in the cochlea. High gene expression was observed in ganglion neurons, glial cells, and supporting cells; however, the vector failed to infect sensory cells. Although lentiviral vectors offer long term gene expression in a variety of cell types, they are known to have a high risk of evoking a strong immune response and generating a replication-competent virus [86].

Currently, no ideal vector exists for use in inner ear gene delivery. However, adenovector based gene therapy is currently the most widely used form of gene therapy in the inner ear. Newer recombinant forms of adenoviruses carrying the *Math1* gene have shown promising results in generating hair cells in both auditory and vestibular systems. Among all known viral vectors, adenovirus is widely used in delivering specific genes (*Math1*, GDNF, and Bcl-2) for regenerating and protecting hair cells. Table 3 lists the advantages and disadvantages of using different viral vectors in inner ear gene therapy.

The development and use of different viral vectors in inner ear gene therapy allows us to evaluate the effects of introducing specific genes and therapeutic molecules that can regenerate hair cells and prevent hair cell damage.

**Table 3:** Summary of different viral vectors used in inner ear gene therapy.

**Vector**	**Advantages**	**Disadvantages**	**Ref.**
**Adeno virus**	1) Transfect a wide variety of cell types in the inner ear including spiral ganglions, outer hair cells, spiral ligament, stria vascularis, and mesenchymal cells in both auditory and vestibular systems. 2) Can produce high titer values that allow injection of small dose volumes for gene therap. 3) Transgene expression up to 3 weeks can be achieved. The short duration of gene expression is ideal for hair cell regeneration because prolonged *Math1* expression can produce too many ectopic hair cellsand compromise hearing. 4) Effective in delivering *Math1* gene to regenerate hair cells and Bcl-2 to protect hair cells from damage. 5) Allows insertion of large DNA segments. Recombinant forms can take up to 30kb foreign DNA. 6) Infects dividing and non-dividing cells with very high transduction efficiencies, both *in vitro* and *in vivo*. 7) Widely researched in clinical studies in both animals and humans, giving a better ability to tackle clinical complications that can arise.	1) Evokes a strong host immune response. 2) Does not offer long term gene expression. 3) Entry of the viral vectors is largely dependent on a host receptor called the coxsackie virus receptor (CAR).	[49,51, 82,83,84,87,88]
**Adeno-associated Virus**	1) Effectively transfect most inner ear cell types *in vivo*. Studies show transgene expression in cochlear blood vessels, nerve fibers and spiral limbus cells. 2) Effective in targeting stria vascularis and delivering tropic factors like NT-3, BDNF, VEGF and FGF. 3) Transgene expression can occur up to 24 weeks *in vivo*. 4) Non-toxic to inner ear cells and evokes low immune response.5) Lacks pathogenicity and has never been associated with any known human disease making them suitable for clinical applications.	1) Only effective with *in vivo* inner ear gene therapy. 2) Successful transgene expression *in vivo* depends on route of vector administration, limited to only direct injection of vector. 3) Previous studies haveshown possible dissemination of vector from target tissue. 4) Offers only a limited payload capacity owing to its small size. 5) Risk of insertional mutagenesis. 6) No substantial clinical experience. 7) Vector entry in to host largely depends on heparin sulfate receptor.	[76,89,90,91,92]
**Herpes Simplex Virus**	1) Effectively known to target non-dividing cells, specific to nerve cells, spiral ganglion, vestibular ganglion and mesenchymal cells in mice and guinea pigs. 2) Newer recombinant vectors offer stable and long term gene expression of up to 8 weeks. 3) Can take large DNA fragments.	1) Evokes a strong immune response. 2) Transfection is limited only to non-dividing neuronal cells. 3) Large size of the virusmakes it difficult tomanipulate. 4) The virus does not integrate into the host genome; hence gene expression can be unstable. 5) Difficult to produce high titer values and requires injection of high vector volumes. 6) No substantial clinical experience.	[84,85,93]
**Lentivirus**	1) Transfect both dividing and non-dividing cells, including stem cells that are very difficult to transfect. 2) Effectively transduce spiral ganglion neurons and supporting cell *in vitro*. 3) Studies indicate transgene expression in perilymphatic space for up to 2 weeks.	1) Limited to use in production of genes only in the perilymph. 2) Limited dissemination of vector and not suitable for sensory cell transduction. 3) Failure to transduce cells in the sensory epithelium *in vivo*. 4) Can randomly integrate into host chromosome and capable of generating a replication competent virus. 5) No clinical experience and safety concern arise from human immune deficiency virus origin.	[86,94]

### 4.2. Stem Cell-Based Therapy for Inner Ear Hair Cell Regeneration

In situations where the loss of supporting cells may prevent transdifferentiation based regeneration strategies, hair cells may be replaced by stem cell therapy. In the last decade, there has been a considerable amount of attention directed toward stem cell-based therapies for treating diseases like Alzheimer's, Parkinson's and cardiovascular diseases. The success of stem cells in treating these diseases opened opportunities for researchers to explore the use of stem cells in treating hearing disabilities. Stem cell therapy is based on the concept that, upon transplantation, the undifferentiated stem cell has the capacity to respond and react to surrounding cell signals and differentiate into the appropriate cell type associated with the signal. Stem cells are a useful way of exploring the molecular pathways that underlie hair cell genesis. Some of the stem cell-based therapeutic strategies that have employed stem cells in the effort to cure hearing loss are listed below.

#### 4.2.1. Embryonic Stem Cells

ESCs are pluripotent and capable of giving rise to cells from any of the three germ layers [95]. With respect to hair cell regeneration, it was reported that murine embryonic stem cells can generate inner ear progenitors *in vitro* [96]. These ESCs were allowed to form embryoid bodies and were cultured in the presence of epidermal growth factor (EGF), insulin-like growth factor 1 (IGF-1), and basic fibroblast growth factor (bFGF). The newly generated progenitor cells were reported to express markers characteristic of hair cell differentiation and hair cell specific markers. Additionally, the progenitor sensory cells had the capacity to integrate into sensory epithelial layers when injected into the developing inner ear of a chicken and to express hair bundle markers *in vivo*. Another study demonstrated the use of murine ESCs from transgenic *Math1*/nGFP mice [97]. The ESCs were differentiated using a step-by-step method toward the ectodermal lineage using otic-inducing growth factors. The generated otic progenitor cells had the capacity to develop into mechanosensitive sensory hair cells *in vitro* and demonstrated immature hair cell transduction currents [97]. ESCs have been reported to produce sensory auditory neurons and neural progenitors with the potential to restore auditory function by generating nerve connections to hair cells [98,99,100]. Embryoid bodies from murine ESCs co-cultured with hair cell explants showed neuron-like cells and positive staining for neurofilament [99]. Most of the embryonic stem cell studies have used murine stem cells; however, there have been attempts to differentiate human embryonic stem cells (hESCs) in the presence of growth factors such as neurotrophin-3 (NT-3), bFGF, BDNF, EGF and bone morphogenic protein 4 (BMP4). These differentiated cells expressed inner ear and synaptic markers [101,102,103]. hESCs have also been cultured to generate otic progenitors that were capable of differentiating into auditory sensory neurons [102].

Although these studies have demonstrated successful *in vitro* and *in vivo* generation of replacement hair cells and auditory neurons from ESCs, further investigations are crucial in developing treatment strategies for hearing loss because of the controversial and ethical issues linked to ESCs.

#### 4.2.2. Adult Stem Cells

Promising results have been shown with bone marrow mesenchymal stem cells (BMSCs) in the field of hair cell regeneration. Mesenchymal stem cells derived from rat bone marrow have been reported to differentiate into inner ear progenitors and express sensory cell markers including myosin VIIa, espin, Brn3c, p27kip and jagged2 *in vitro* [104]. Additionally, the differentiated cells displayed morphological characteristics of hair cell stereociliary bundles. Studies have shown that BMSCs stimulated in the presence of growth factors were able to form neuronal progenitors, and after being transfected with the *Math1* gene, were able to differentiate into inner ear sensory-like cells [104]. Adult stem cells also have the potential to deliver gene and therapeutic molecules to other parts of the inner ear. For example, Connexin 26, a protein present in cochlear gap junctions and supporting cells was expressed when bone marrow stromal cells were transplanted into the perilymphatic space of the mouse cochlea [105]. Bone marrow mesenchymal stem cells also have the potential to differentiate into auditory neurons *in vitro* and *in vivo* [106,107], demonstrating that a wide variety of inner ear cell types can be generated from stem cells.

Adult stem cells isolated from mouse macular organs have been shown to differentiate into hair cells when cultured with EFG and IGF-1 [108]. Adult stem cells isolated from olfactory neuroepithelium expressed hair cell markers and resembled hair cells phenotypically when co-cultured with cochlear cell supernatant [109].

Transplantation studies in mice with bone marrow-derived hematopoietic stem cells (BMHSCs) suggested the possibility of differentiation of BMHSCs into mesenchymal cells and fibrocytes in the adult inner ear [110]. The results with BMHSCs show their potential to attenuate cochlear injury by replacing mesenchymal cells and fibrocytes in the inner ear [110].

Adult stem cells from many tissues are now being used to investigate cures for various diseases. These cells are not concerned with any ethical issues and can enter clinical trials involving autologous transplantation therapies and used in bioengineered products. In treating inner ear disorders, bone marrow-derived stem cells have shown the most favorable results [105,106,110,111].

#### 4.2.3. Induced Pluripotent Stem Cells

The discovery of generating induced pluripotent stem cells (iPSCs) introduced a new dimension to stem cell research. iPSCs are produced from fibroblasts, other somatic cells and adult stem cells, which are reprogrammed to express certain genes and maintain characteristic properties of embryonic stem cells. iPSCs opened up the possibility of generating and using patient-specific stem cells without immune rejection *in vivo* and, unlike hESCs, there are no controversial issues associated with their use. The most important factors in maintaining the pluripotency of ESC lines are *Oct4*, *c-Myc*, *Klf4* and *Sox2*. Any adult stem cells forced to express the above four genes under ESC culture conditions can be reprogrammed into ESC-like cells [112]. Recently, it was demonstrated that human neural stem cells can be directly reprogrammed to iPSCs by just expressing *Oct4* [113]. Like ESCs, most studies with iPSCs in hair cell regeneration are also of murine origin. A recent study showed generation of iPSCs from murine embryonic fibroblasts [97]. These fibroblasts were transduced with retroviruses to express *Oct4*, *c-Myc*, *Klf4* and *Sox2*. The generated iPSCs were cultured in a medium containing otic-inducing FGF-3 and FGF-10 to produce otic progenitor cells. The generated otic progenitors differentiated into hair cell-like cells expressing hair cell markers. When co-cultured with fibroblast-like cells from embryonic chicken utricles, the differentiated cells developed hair bundle-like protrusions, responded to mechanical stimulation, and displayed transduction currents [97]. Another study explored the use of iPSCs for restoring auditory ganglion neurons. *In vitro* neuronal differentiation of iPSCs was induced by exposing them to stromal cell-derived inducing activity (SDIA). SDIA is a neural inducing activity demonstrated in stromal cells when they simultaneously produce inducing and inhibitory factors and was developed by Kawasaki *et al.* [114]. The differentiated cells were transplanted into the cochleas of mice. iPS cell-derived neurons projecting toward cochlear hair cells were observed 1 week after transplantation [115]. Although iPS cell techniques are novel, researchers have started to explore their potential in treating SNHL.

#### 4.2.4. Summary of Stem Cell-Based Therapies

ESCs have high survival rates and migration capacity when implanted into the cochlea [116,117]. They migrated onto auditory neurons and exhibited neuronal differentiation. However, ESCs exhibited low integration into endogenous tissue and failed to differentiate completely at the implantation site [118,119]. There is also the risk of tumor formation and the risk of transmitting infections with ESCs because they use animal products during the culturing process. ESCs used in inner ear treatment require the use of immunosuppressive therapy or cloning to avoid graft-vs-host diseases. On the other hand, adult stem cells isolated from bone marrow can easily bypass the immune barriers and overcome the problem of immune rejection. BMSCs also have a high survival rate and can migrate into multiple regions in the cochlea and brain [106,120,121,122]. Although they can be stimulated to differentiate into a number of cell lineages, they differentiate more toward the mesoderm lineage and can be used to replace degenerated cochlear fibrocytes [123]. iPSCs do not have any ethical concerns with their use and are more patient specific, thus eliminating the risk of immune rejection. Their undifferentiated state allows them to migrate to regions surrounding the cochlea. However, one of the major concerns with iPSCs is the time required to produce the individual cell lines [124]. They also pose the risk of passing on the DNA from the genetically altered cell to future generations.

Some of the barriers that exist with the use of stem cells are the formation of tumors and graft-versus-host diseases. Another concern is the integration of stem cells in the inner ear. Owing to the spiral structure of the cochlea, it is challenging to direct the stem cells into the desired location and evaluate the integration of differentiated stem cells inside the cochlea. Further investigation with the use of different stem cell types and advancements in the existing approaches are necessary before they can enter the clinic and begin treating hearing disorders.

Nevertheless, these concerns bring opportunities for bioengineers and clinicians to engineer cells and vectors carrying a combination of genes and therapeutics and develop methods and devices to deliver therapeutics at appropriate locations. Recent advances in stem cell technology and gene based therapies provide the framework required for the development of potential treatment options.

## 5. Discussion

The success of current approaches unveils the possibility of using different viral vectors, genes, growth factors and stem cell types in regenerating inner ear hair cells. Nevertheless, more research is still required to overcome challenges involved before these approaches will be ready for clinical and commercial use. Although several studies have reported the generation of hair cell-like cells *in vitro* and *in vivo* (in animals)*,* the outcome and characteristic properties of these cells must be further explored. A cell that simply resembles a hair cell morphologically and expresses hair cell markers would be immature without being able to respond to mechanical stimuli and transfer signals to the auditory neurons. A detailed investigation of their ultra structure and ability to connect to auditory neurons and produce transduction currents is essential. In terms of functional outcomes, crucial to any tissue engineering strategy, the mechanoelectrical transduction and maturity of transplanted hair cell-like cells can be evaluated by auditory brainstem response (ABR) and otoacoustic emissions tests (OAE) in animal studies.

Audiologists and scientists have come a long way toward reaching the goal for hair cell regeneration in mammals by developing inner ear gene therapy strategies in animal models. However, an improvement in the existing gene delivery techniques is required to suit clinical applications.

Some of the drawbacks associated with inner ear gene therapy are addressed below. For example, gene delivery studies have shown that over-expression of the *Math1* gene led to the formation of ectopic hair cells [16,50]. Hair cells are extremely rare and the cochlea contains only about 14,000 hair cells that detect and amplify sound [103], and it is important to regulate the cell cycle for normal development of cochlear function. Studies in p27kip knockout mice resulted in production of supernumerary hair cells and later a massive degradation of the hair cells occurred, leading to a severe hearing impairment in these animals. This indicates the importance of precise control of cell cycle [69]. There are several pathways that control the expression of *Math1* and drive a cell toward differentiating into a hair cell. *Math1* inhibitors and down regulators can be used in addition to targeting the inner ear with only the *Math1* gene. This approach can be used to produce an appropriate number of hair cells. The activation of a notch receptor can up regulate *Hes* and *Hey* genes that are potent inhibitors of *Math1* [18,19,57]. Inclusion of notch receptor inhibitors in gene therapy can help in boosting *Math1* gene expression. However, a balance of expression and inhibition between the *Math1* gene and *Math1* inhibitor is required for normal inner ear development and hearing.

Besides developing strategies to control the expression of the *Math1* gene, the side effects caused by viral routes also need to be considered. Although viral methods have shown tremendous success in the animal model, there is always the risk of DNA mutations and cancer associated with viruses. Non-viral gene delivery techniques have recently gained attention due to the minimal risks associated in terms of clinical safety and reliability. Another drawback of *Math1* gene therapy is that although it can be a potential cure for hearing loss caused by sound trauma or ototoxic damage, it may not cure hearing loss caused by genetic defects. Stem cell-based therapies *in vivo* have reported migration of these cells into different areas, and thus an ideal route for delivering cells to the right location in the cochlea is crucial to restoring hearing loss. The large majority of studies have used only animal models and animal stem cells. There is no doubt that these studies have played a major role in gathering valuable information about hair cell regeneration; however, the variation among species requires the need to explore the use of human stem cell lines like bone marrow derived mesenchymal and hematopoietic stem cells, umbilical cord blood and umbilical mesenchymal stromal stem cells, and induced pluripotent stem cells. Research in human stem cells lines will have more clinical relevance.

Gene delivery and stem cells are a potential cure to hearing loss; however, there are limitations that must be overcome. Gene therapies must consider using combinations of essential genes and cell cycle inhibitors to control the production of hair cells, and detailed quantification of hair cell functionality is necessary. Gene delivery vehicles with minimal risk of mutagenesis and immune response must also be developed. Finally, an ideal route to implant stem cells inside the cochlea would be essential for successful innervations of hair cells, thereby allowing better, if not pristine transmission of sound with the assistance of a cochlear implant.
